# Shift one's trouble to others: Does climate policy uncertainty promote enterprises' “pollution migration” in the context of artificial intelligence?

**DOI:** 10.3389/fpubh.2025.1578139

**Published:** 2025-09-10

**Authors:** Jianming Wang, Wei Li

**Affiliations:** ^1^School of Management, Zhejiang University of Finance & Economics, Hangzhou, China; ^2^The New Types Key Think Tank of Zhejiang Province “China Research Institute of Regulation and Public Policy”, Zhejiang University of Finance & Economics, Hangzhou, China

**Keywords:** climate policy uncertainty, pollution migration, artificial intelligence adoption, financing constraints, pollution haven

## Abstract

Against the backdrop of worsening global climate change, countries worldwide have implemented climate policies to reduce corporate pollution emissions and promote corporate social responsibility. However, regional differences in climatic conditions have intensified the uncertainty of climate policies during implementation, creating a critical research gap: the influence of climate policy uncertainty (CPU) on corporate pollution behavior remains underexplored, despite its theoretical value for enriching environmental policy and corporate behavior research and practical significance for guiding policy optimization. To address this gap, this study takes 3,702 listed enterprises across 31 provinces in China (2010–2022) as the research sample. It empirically examines the impact of CPU on enterprises' “pollution migration” behavior, with a focus on testing underlying mechanisms (e.g., financing constraints) and heterogeneous effects (e.g., by artificial intelligence [AI] adoption level, enterprise pollution intensity, and ownership type). The key findings are as follows: (1) CPU significantly exacerbates enterprises' pollution migration; (2) the mechanism test confirms that CPU increases enterprises' financing constraints, which in turn aggravates pollution transfer; (3) enterprises with higher AI adoption levels experience a weaker impact of CPU on pollution migration; and (4) heterogeneity analysis shows that CPU exerts a more pronounced effect on pollution migration among highly polluting enterprises and non-state-owned enterprises (NSOEs). This study validates the “pollution haven” hypothesis in the context of climate policy uncertainty, providing important references for both policymakers and enterprises. For governments, it is recommended to stabilize climate policy expectations, improve the green financial system, and support enterprises in AI application. For enterprises, proactive monitoring of policy trends and enhancement of AI application capabilities are essential to mitigate the adverse effects of CPU and achieve sustainable development.

## 1 Introduction

In the current context of the increasingly severe global climate situation, the challenges posed by climate change have become the focus of common concern for all mankind. Against this backdrop, governments around the world have introduced a series of climate policies to address the worsening environmental issues and achieve global climate goals (1). Although climate policies can reduce enterprises' carbon emissions and achieve green development (2). However, they face numerous problems during the implementation process. Among them, the frequent adjustment and uncertainty of policies have become significant challenges for enterprises' business decisions (3). The uncertainty of climate policies stems from various factors, such as changes in the international political situation, the game among different interest groups, and the continuous deepening of understanding of climate science. This uncertainty poses many difficulties for enterprises when making pollution control and investment decisions (4). The enterprises find it difficult to accurately predict future environmental regulatory standards and policy costs, which directly affects their production and operation plans (5).

In recent years, with the increasing uncertainty of the climate, the effectiveness of climate policy implementation has also been accompanied by growing uncertainty (80). In the case of China, the lack of a unified measurement framework and a clear definition of climate policies has led to a higher level of climate policy uncertainty (CPU) among local governments (6). A growing number of studies have shown that the uncertainty of climate policies not only affects enterprises' investment decisions but may also prompt enterprises to avoid policy risks by transferring pollution (7). To cope with policy uncertainty, enterprises may choose to relocate polluting activities to regions with more lenient regulations, thus exacerbating environmental inequality among regions (8). Moreover, against the backdrop of the rapid development of artificial intelligence, artificial intelligence (AI) technology has had a profound impact on enterprises' operational and risk management capabilities (9, 10). Artificial intelligence technology, with its powerful data analysis, prediction, and automation capabilities, is transforming enterprises' production models, external risk response capabilities, and environmental protection behavior decisions (11). The enterprises with different levels of artificial intelligence adoption demonstrate varying abilities and strategies when facing external environmental changes (12).

While previous studies have delved into the influence of economic policy ambiguity on enterprises' “pollution migration” actions (13), investigations into how climate policy alterations impact enterprises' pollution conduct are still scarce (14). The swift evolution of artificial intelligence is certain to modify the extent to which climate policy shifts affect enterprises' pollution behavior, and this has emerged as a crucial element affecting the sustainable development of enterprises. Nevertheless, at present, no research has zeroed in on the impact mechanism of diverse levels of information asymmetry on pollution transfer behavior in enterprises with different degrees of artificial intelligence implementation. Additionally, when it comes to the measurement metrics of climate policy uncertainty, numerous Chinese scholars utilize the climate policy uncertainty index computed by Gavriilidis (15) on the basis of major American newspapers (16). Consequently, this research makes the following marginal contributions: firstly, in light of the actual circumstances in China, this study computes the climate policy uncertainty for different cities in China and conducts empirical examinations. Secondly, this study gauges the financing constraints confronted by Chinese enterprises based on the four indices of FC, SA, WW, and KZ, and elucidates the potential routes through which climate policy uncertainty influences enterprises' “pollution migration” behavior. Finally, this study integrates the degree of AI adoption into the analytical framework of climate policy uncertainty and enterprises' pollution migration.

## 2 Research hypotheses and theoretical foundations

### 2.1 Impact of climate policy uncertainty on enterprises' pollution transfer

As the main body of economic activities, enterprises' production and operation activities inevitably generate emissions of pollutants such as wastewater, waste, and emissions gases (17). In an environment where climate policies are relatively stable and strict, enterprises will increase their investment in emission reduction, optimize production processes, and reduce pollution emissions in order to meet the environmental emission reduction thresholds of local governments or obtain government environmental protection subsidies (18). Environmental policies can exert a significant impact on enterprises' carbon emissions and the economic growth of local regions (19). However, when there is uncertainty in climate policies, the effectiveness of the government will decrease accordingly (20), a phenomenon that aligns with Bellassen and Shishlov's (21) exploration of pricing monitoring uncertainty in climate policy. As an important external risk faced by enterprises, the uncertainty of climate policies will increase policy risks, thereby enhancing the impact on enterprises' external risks. The expected risks and returns faced by enterprises will change (22, 23), which echoes Morão's (24) findings on the influence of climate policy uncertainty on the energy industry. For enterprises, the uncertainty of climate policies makes it more difficult for them to accurately predict future climate policy costs (25), such as the implementation details and intensity of policy tools like carbon taxes and emission quotas. This makes enterprises more cautious in their environmental protection investment decisions (26, 27), and relates to Attílio's (28) research on how such uncertainty spills over to affect green innovation behaviors. In this case, enterprises may be inclined to transfer pollution intensive production processes to regions with relatively lenient climate policies or weaker regulatory efforts (29) to reduce the environmental cost risks they face, thus exacerbating pollution transfer behavior (30), a dynamic that Golub et al. (31) indirectly highlight when discussing the need to escape climate policy uncertainty traps.

On the other hand, the existence of climate policy uncertainty may also affect the market competition pattern of enterprises (32), which is consistent with Ayed et al.'s (33) observation that climate policy uncertainty influences corporate dividend strategies, reflecting broader impacts on business decisions. Some enterprises may take advantage of the differences in climate policies among different regions to obtain cost advantages through pollution transfer, thereby strengthening their position in market competition (34). For example, when climate policies in some regions strictly restrict the development of highly polluting industries while the policy implementation in other regions is weak, enterprises may transfer polluting production activities to the latter to obtain a higher profit margin (35). This behavior not only leads to the transfer of pollution among regions but may also trigger the “pollution haven effect” (36), that is, enterprises choose to carry out production activities in regions with lenient environmental policies to avoid strict environmental supervision (37), a concern that Meah (38) touches upon when examining policy makers' understanding of climate uncertainty's implications.

In addition, the uncertainty of climate policies can significantly affect enterprises' enthusiasm for green technology innovation (39). When the local governments have a relatively long cycle for climate policy adjustments, enterprises are more inclined to invest more funds or other resources in green technology innovation to ensure compliance with the government set environmental protection criteria (40). However, when the policy uncertainty is high, enterprises may worry about the risks of investing in green technologies (15), thus reducing their investment in the research, development, and application of environmental protection technologies (26), which in turn relates to Kim et al.'s (41) findings on how climate policy uncertainty affects corporate environmental risk-taking. In this case, enterprises are more likely to rely on existing production technologies and models and cope with short term environmental cost pressures through pollution transfer instead of fundamentally changing their production methods to reduce pollution emissions (42). Therefore, this study proposes Hypothesis 1:

H1: Climate policy uncertainty will exacerbate enterprises' pollution transfer behavior.

### 2.2 The mediating role of financing constraints

The uncertainty of climate policies will increase the emission reduction costs of polluting enterprises, which in turn will increase the financing costs of enterprises (34). In recent years, the uncertainty of climate policies, regarded as a government-related risk, has become one of the important and inevitable external risks in the process of enterprise operations (81). This uncertainty not only affects enterprises' daily operational costs but also disrupts their formulation of long-term green strategies, as fluctuating policy expectations make it difficult for enterprises to invest stable funds in green governance (43). As governments around the world pay increasing attention to environmental protection, they have successively introduced more and more stringent climate policies. Stakeholders such as enterprise management teams and investors have also started to incorporate climate policies into the future strategic planning of enterprises. The perceptions and preferences of stakeholders like enterprise management teams and investors regarding climate policies can significantly influence the future strategic planning of enterprises (82, 83). Third-party financial institutions such as commercial banks and securities institutions also take into account the impact of climate policies on enterprise operations and conduct regular assessments of the expected future climate risks of enterprises, similar to how Gounopoulos and Zhang (44) note that environmental factors like temperature trends affect corporate financial decisions. This has led to the impact of climate policy uncertainty on the financing constraints of enterprises (83).

When the uncertainty of climate policies is high, the expected future operating risks and costs of enterprises will increase accordingly. Financial institutions such as commercial banks and securities enterprises usually conduct regular assessments of the expected future risks of enterprises. They take a more cautious attitude toward enterprises with higher expected future risks and will raise the financing threshold or increase the financing costs for such enterprises (23), a dynamic that echoes Aglietta et al.'s (45) exploration of climate finance challenges. This makes it easier for enterprises to fall into the dilemma of financing constraints when facing climate policy uncertainty (46). Moreover, emission reduction and green technology innovation are characterized by high risks, low returns, and long payback periods. With the increase in financing constraints, enterprises' capital investment in emission reduction and green technology innovation will be greatly reduced (47), which is consistent with Ghisetti et al.'s (48) research on financial barriers limiting environmental innovations. Regional differences in policy enforcement intensity, amplified by uncertainty, result in uneven environmental cost pressures across regions. This disparity further incentivizes enterprises to seek cost-saving alternatives such as pollution transfer (49), a phenomenon that can be linked to Narita et al.'s (50) insights on how economic measures in climate interventions influence regional disparities. Therefore, when enterprises face high financing constraints, their green transformation strategies will be restricted. In this case, enterprises may choose to transfer pollution intensive production processes to regions with weaker environmental supervision or more lenient climate policies to reduce short term environmental cost pressures, thus exacerbating pollution transfer behavior (84). In addition, financing constraints may also affect enterprises' strategic decisions. When facing a shortage of funds, enterprises tend to choose short term survival strategies rather than long term sustainable development strategies. Pollution transfer behavior can bring cost advantages to enterprises in the short term and enhance their position in market competition. Therefore, this study proposes Hypothesis 2:

H2: Climate policy uncertainty will increase the financing constraints faced by enterprises, thereby exacerbating enterprises' pollution transfer behavior.

### 2.3 The moderating role of the adoption level of artificial intelligence

The degree of artificial intelligence adoption can significantly enhance enterprises' operational efficiency and decision making quality. Through data analysis, machine learning, and automated processes, enterprises can more accurately predict market demands, optimize production processes, reduce operating costs, and improve resource utilization efficiency (51). This improvement in efficiency not only helps enterprises maintain their competitiveness in an uncertain environment but also enhances their financial performance, thereby improving their credit ratings and financing capabilities in the financial market (52, 53). Therefore, compared with enterprises with a low degree of artificial intelligence adoption, enterprises with a high degree of artificial intelligence adoption face less risk when financing constraints change (54, 55). On the one hand, according to the information asymmetry theory, for third party financial institutions such as commercial banks and securities enterprises, enterprises with a high degree of artificial intelligence adoption have higher quality and more transparent information disclosure. These enterprises have a lower degree of information asymmetry, and the disclosed information is more reliable (56). Therefore, banks can assess the repayment ability of enterprises through information such as financial statements, public opinion information, and consumer sentiment disclosed by enterprises, and thus provide loans with lower costs and lower thresholds for enterprises, alleviating the financing constraints of enterprises and reducing the impact of climate policy uncertainty on enterprises' financing constraints (57). On the other hand, the degree of artificial intelligence adoption can also improve enterprises' investment efficiency (58) and green innovation capabilities (59), thereby reducing enterprises' carbon costs (60) and decreasing enterprises' pollution transfer behavior (52). Through artificial intelligence technology, enterprises can optimize production processes, reduce production costs, and improve risk bearing capabilities, thereby enhancing enterprises' energy efficiency (61). Moreover, enterprises with a high degree of artificial intelligence adoption face less severe financing constraints. This enables enterprises to maintain a certain level of pollution control capabilities through their technological and cost advantages when facing financing constraints, reducing the motivation for pollution transfer (62, 63). In the impact mechanism of climate policy uncertainty on enterprises' pollution transfer behavior, the degree of artificial intelligence adoption plays a certain mitigating role (62, 63).

Therefore, this study proposes the following Hypothesis 3:

H3: The degree of artificial intelligence adoption can alleviate the impact of climate policy uncertainty on enterprises' financing constraints.

### 2.4 The impact of climate policy uncertainty on the pollution transfer of enterprises with different industry and ownership

In the impact mechanism through which climate policy uncertainty influences enterprises' pollution transfer behavior, the heterogeneity in enterprise types and industry characteristics can lead to remarkably different outcomes (64). For one thing, owing to the heavily pollution emission features of their production processes, heavily polluting enterprises usually encounter more stringent environmental supervision and higher costs for emission reduction. When climate policy uncertainty is high, compared with non-heavily polluting enterprises, heavily polluting and energy intensive enterprises are confronted with more substantial policy risks (65). Heavily polluting enterprises are required to allocate more resources to pollution control and emission reduction measures in order to meet the increasingly strict environmental standards. The uncertainty of climate policies renders it challenging for heavily polluting enterprises to precisely forecast future policy requirements and compliance costs, thereby escalating their operational risks and cost expectations (66).

On the other hand, compared with central SOEs and local SOEs, climate policy uncertainty has a greater impact on the pollution transfer behavior of non-state-owned enterprises (NSOEs) (67). Against the special background of China, NSOEs are usually at a disadvantage compared with SOEs in terms of resource acquisition, policy support, and financing channels. When facing climate policy uncertainty, since state owned enterprises have the government as a credit guarantee, financial institutions set lower financing constraint thresholds and costs for SOEs, while NSOEs face higher financing constraint thresholds and costs. In addition, non-state owned enterprises have lower flexibility in coping with policy risks, and their operating risks and cost expectations are more easily affected by climate policy uncertainty (68). In this case, NSOEs are more likely to adopt pollution transfer behavior to reduce short term costs and risks and maintain their competitiveness. Finally, executives of SOEs and NSOEs may have varying degrees of overseas experience. For NSOEs, if their executives lack overseas experience and exposure to more advanced environmental management concepts, their ability to formulate effective strategies to cope with climate policy uncertainty may be relatively weak. In contrast, executives of SOEs may have more opportunities to access diversified resources and international experience, which enables them to better guide enterprises in making internal adjustments rather than resorting to pollution transfer (69). In contrast, SOEs, due to enjoying more policy support and resource guarantees, may be more inclined to adopt internal adjustments rather than pollution transfer when dealing with climate policy uncertainty (70). Therefore, the following hypotheses are proposed in this study:

H4a: The impact of climate policy uncertainty on the pollution transfer behavior of heavy polluting enterprises is more significant.
H4b: The impact of climate policy uncertainty on the pollution transfer behavior of non-state owned enterprises is more significant.

## 3 Data and empirical analysis

### 3.1 Sample selection and data source

Since the climate policies of various provinces in China were gradually issued after 2008, and the relevant data on climate policies disclosed by each province were basically gradually improved after 2010. Therefore, this study selects the period from 2010 to 2022 as the research time frame. After excluding enterprises that were delisted or on the verge of delisting in the past 2 years, this study has collected relevant data from 3,702 listed enterprises in provinces and autonomous regions of China. Finally, this research obtained panel data of 33,274 sample observations, and all the data were logarithmically transformed and winsorized at the 1 and 99% levels.

### 3.2 Variable definition

#### 3.2.1 Climate policy uncertainty

This study draws on the climate policy uncertainty index constructed by Ma et al. (71) to measure the degree of local climate policy uncertainty for enterprises. This indicator uses the MacBERT model to conduct text analysis on a total of 1,755,826 news articles from mainstream Chinese newspapers and periodicals. The data is processed through multiple steps, including data cleaning, manual verification, and model construction, ultimately resulting in the construction of China's provincial CCPU indices from 2010 to 2022, which cover 31 provinces. This method can effectively avoid objectivity issues in the construction of climate policy uncertainty indicators.

#### 3.2.2 Enterprises' pollution transfer

This study draws on the methods for constructing the geographical center of gravity proposed by Li et al. (35) and Chen et al. (72) to measure the pollution transfer of enterprises through a spatial weight matrix. Assume that the coordinate of an enterprise is (*x*_*i*_, *y*_*i*_), and the specific calculation method is as follows:


$$\begin{array}{l} {\text{x}}_{\text{i}}=\frac{\sum_{\text{i}=1}^{\text{n}}{\text{x}}_{\text{i}}{\text{w}}_{\text{i}}}{\sum_{\text{i}=1}^{\text{n}}{\text{w}}_{\text{i}}},\;{\text{y}}_{\text{i}}=\frac{\sum_{\text{i}=1}^{\text{n}}{\text{y}}_{\text{i}}{\text{w}}_{\text{i}}}{\sum_{\text{i}=1}^{\text{n}}{\text{w}}_{\text{i}}} \end{array}$$

Where:

x_i_: the longitude of the city. When i = 1, 2, 3, they respectively represent the longitude of the pollution center of gravity, the longitude of the environmental regulation center of gravity, and the longitude of the economic development center of gravity of the enterprise.

y_i_: the latitude of the city. When i = 1, 2, 3, they respectively represent the latitude of the pollution gravity, the latitude of the environmental regulation gravity, and the latitude of the economic development gravity.

w_1_: the weight of the pollution gravity. In this study, it is measured by the total amount of pollution emission equivalents of enterprises in the region.

w_2_: the weight of the environmental regulation gravity. In this study, it is measured by the proportion of investment in pollution control by enterprises in the region in the current year.

w_3_: the weight of the economic development gravity. In this study, it is measured by enterprises' ROA.

The specific calculation method for the pollution transfer distance of enterprises is as follows:


$$\begin{array}{l} DIS_{it}={\left[{\left((\frac{y_{2}+y_{3}}{2})-y_{1}\right)}^{2}+{\left((\frac{x_{2}+x_{3}}{2})-x_{1}\right)}^{2}\right]}^{1/2} \end{array}$$

The greater the deviation degree *DIS*_*it*_ between the “economic development—environmental regulation” gravity and the “pollution” gravity of an enterprise, the higher the degree of the enterprise's pollution transfer.

#### 3.2.3 Financing constraints

This study draws on the financing constraint indicators constructed by previous scholars to measure the financing constraints, and constructs the financing constraint FC index, SA index, WW index, and KZ index for listed enterprises, respectively. Among them, the financing constraint FC index is formed by standardizing variables such as enterprise scale, age, and cash dividend payment ratio, and then using the Logit model to measure the financing constraint of the enterprise for each year (73). The SA index is mainly obtained by regressing the total assets and the total age to measure the enterprise's financing constraint SA index (74). The WW index is mainly calculated based on the enterprise's earnings before interest and taxes and capital expenditures to obtain the enterprise's financing constraint (75). The KZ index is obtained by regressing the net operating cash flow and Tobin's Q of the enterprise in each year to measure the enterprise's financing constraint KZ index (76).

#### 3.2.4 Artificial intelligence (AI) adoption

The degree of artificial intelligence (AI) adoption by enterprises is measured using a comprehensive index constructed based on the frequency of AI-related keywords in corporate annual reports and the proportion of investment in AI technologies. Specifically, this study draws on the methods of Li et al. (11) and Sun et al. (10). First, Python is used to conduct text analysis on the annual reports of listed companies to identify core keywords such as “artificial intelligence,” “machine learning,” “big data analytics,” “deep learning,” and “intelligent algorithms.” Subsequently, the frequency of these keywords is counted and standardized to reflect the attention and disclosure intensity of enterprises regarding AI applications. Meanwhile, the proportion of R&D investment in AI-related fields (such as procurement of intelligent equipment, training of AI talents, and cooperation with AI technology enterprises) in the total R&D investment of enterprises is incorporated as a supplementary indicator to measure the actual investment intensity in AI technologies. Finally, these two types of indicators are weighted and combined to form a comprehensive index of the degree of AI adoption (denoted as AI). A higher index value indicates a higher level of AI application by the enterprise. This measurement method not only considers the emphasis on AI in the textual expressions at the strategic level of enterprises but also reflects the actual resource input, thereby more comprehensively and accurately quantifying the degree of AI adoption.

#### 3.2.5 Control variables

This study refers to relevant domestic and foreign literature and selects control variables including enterprise size, financial leverage, effective annual rate, growth ability, years on market, and return on net assets. Enterprise size: this study measures enterprise size using the natural logarithm of the total assets at the end of the enterprise's fiscal year, which reflects the enterprise's resource base and market position. The data is sourced from the balance sheet data of enterprises in the China Stock Market and Accounting Research Database (CSMAR) and Wind Database. Financial Leverage: this study calculates financial leverage as the ratio of total liabilities to total assets of an enterprise, which measures the enterprise's debt risk level. The data is obtained from the liability and asset items in the annual reports of enterprises in the CSMAR Database and Wind Database. Effective Annual Rate: this study calculates the effective annual rate as the ratio of an enterprise's interest expenses to average interest-bearing liabilities, which reflects the enterprise's financing cost. Both interest expenses and interest-bearing liabilities data are derived from the details of enterprise financial expenses in the CSMAR Database and Wind Database. Growth Ability: this study measures growth ability using the operating income growth rate, calculated as [(Current operating income – Previous operating income) / Previous operating income] × 100%, which reflects the enterprise's development potential. The operating income data is sourced from the income statements of enterprises in the CSMAR Database and Wind Database. Years on Market: this study calculates the years on market as the number of years from the enterprise's initial public offering (IPO) year to the sample year, which measures the enterprise's market maturity. The IPO time data is obtained from the company basic information tables in the CSMAR Database and the Wind Database. ROA: this study calculates return on assets as the ratio of net profit to average net assets, which reflects the enterprise's profitability. The net profit and net assets data are sourced from the income statements and balance sheets of enterprises in the CSMAR Database and Wind Database. The data of the above control variables have all undergone winsorization at the 1 and 99% quantiles to avoid the interference of extreme values on the regression results. The data are mainly sourced from the secondary public databases CSMAR and Wind, with some missing values manually supplemented through enterprises' annual reports to ensure the completeness and accuracy of the samples.

The definitions of the variables are shown in the Table 1.

**Table 1:** The definitions of the variables.

**Indicator**	**Specific indicator**	**Symbol representation**
Explained variables	Enterprises' pollution transfer distance	DIS_i, t_
Explanatory variables	Climate policy uncertainty	CPU_i, j, t_
Mediating variables	Financing constraints	FC_i, t_
Moderating variables	Degree of artificial intelligence adoption	AI_i, t_
Control variables	Enterprises size	*SIZE*
	Financial leverage	*LEV*
	Effective annual rate	*EAR*
	Growth ability	*GRO*
	Years on market	*YEAR*
	Return on net assets	*ROA*

### 3.3 Model design

**Figure 1:**
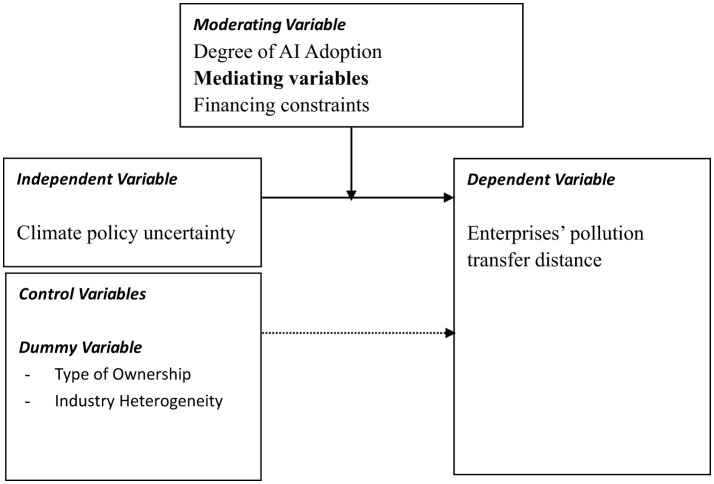
Research framework.

Figure 1 presents the research framework of this study. The dependent variable of this study is corporate pollution migration (measured by pollution transfer distance, DIS), and the independent variable is climate policy uncertainty (CPU). To gain a deeper understanding of the research topic, this paper examines the mediating role of financing constraints (measured by FC, SA, WW, and KZ indices) and also analyzes the moderating role of the degree of artificial intelligence adoption (AI).

In addition, enterprise size, financial leverage, and growth ability are included in the model as control variables, as they are generally believed to have an impact on enterprises' pollution migration behavior. Enterprise size (SIZE) is measured by the natural logarithm of total assets at the end of the fiscal year, reflecting the enterprise's resource base and market position, which may affect its ability to relocate production or invest in pollution control. Financial leverage (LEV) is calculated as the ratio of total liabilities to total assets, used to measure the enterprise's debt risk level; a higher leverage ratio may restrict enterprises' flexibility in responding to policy uncertainty, thereby affecting pollution transfer decisions. Growth ability (GRO) is measured by the operating income growth rate, reflecting the enterprise's development potential. Enterprises with rapid growth may have different motivations for pollution migration compared to those with stable development.

The dummy variables of this study are ownership type and industry type. For ownership type, SOEs are assigned a value of 1, and NSOEs are assigned a value of 0. For industry type, polluting industries are assigned a value of 1, and non-polluting industries are assigned a value of 0. This study has carried out detailed model?? based on the research framework.

First, this study analyzes the relationship between the impact of climate policy uncertainty on the degree of enterprises' pollution transfer by establishing a benchmark regression model as follows:


$$\begin{array}{l} DIS_{it}=\beta_{0}+\beta_{1}CPU_{ijt}+\beta_{2}X_{it}+\mu_{i}+\mu_{t}+\mu_{c}+\varepsilon_{it} \end{array}$$

Where:

*DIS*_*it*_: enterprises' pollution transfer distance

*CPU*_*ijt*_:Climate policy uncertainty

*X*_*it*_:Control Variables

μ_*i*_:Individual control effect

μ_*t*_:Time control effect

μ_*c*_: enterprises' control effect

Second, this study analyzes the mediating role of financing constraints in the mechanism of climate policy uncertainty on the degree of enterprises' pollution transfer by establishing a mediating mechanism test regression model as follows:


$$\begin{array}{r} FC_{it}=\beta_{0}+\beta_{1}CPU_{ijt}+\beta_{2}X_{it}+\mu_{i}+\mu_{t}+\mu_{c}+\varepsilon_{it}\\ DIS_{it}=\beta_{0}+\beta_{1}FC_{it}+\beta_{2}X_{it}+\mu_{i}+\mu_{t}+\mu_{c}+\varepsilon_{it} \end{array}$$

Where:

*FC*_*it*_: financing constraints

Finally, this study analyzes the moderating role played by the degree of AI adoption in the mechanism of climate policy uncertainty on enterprises' finance constraints through the establishment of a regulatory mechanism test regression model as follows:


$$\begin{array}{c} FC_{it}=\beta_{0}+\beta_{1}CPU_{ijt}+\beta_{2}X_{it}+\mu_{i}+\mu_{t}+\mu_{c}+\varepsilon_{it}\\ FC_{it}=\beta_{0}+\beta_{1}CPU_{ijt}+\beta_{2}AI_{it}+\beta_{3}X_{it}+\mu_{i}+\mu_{t}+\mu_{c}+\varepsilon_{it}\\ FC_{it}=\beta_{0}+\beta_{1}CPU_{ijt}+\beta_{2}AI_{it}+\beta_{3}AI_{it}^{*}CCPU_{it}+\beta_{3}X_{it}+\\ \mu_{i}+\mu_{t}+\mu_{c}+\varepsilon_{it} \end{array}$$

Where:

*AI*_*it*_:degree of artificial intelligence adoption.

## 4 Empirical results and analysis

### 4.1 Descriptive statistics

In this study, descriptive statistics of explanatory variable ln(*CPU*), explained variable ln(*DIS*), mediating variables ln(*SA*), ln(*WW*), ln(*KZ*), ln(FC) and moderating variables ln(*AI*), and control variables *ln*(*SIZE*), LEV, *EAR*, *GRO*, ln(*YEAR*), and *ROA* of the selected sample enterprises are presented in Table 2.

**Table 2:** Descriptive statistics.

**Variable**	**Description**	**Obs**	**Mean**.	**Std. Dev**.	**Min**.	**Max**.
ln(*DIS*)	Pollution transfer distance	33,468	5.368	3.403	0.003	23.719
ln(*CPU*)	Climate policy uncertainty	33,468	−0.7359	0.959	−2.308	0.591
ln(*SA*)	Financing constraints SA index	33,468	−3.793	0.290	−5.888	2.131
ln(*WW*)	Financing constraints WW index	33,468	−1.056	25.92	−4.712	0.251
ln(*KZ*)	Financing constraints KZ index	33,468	1.522	2.203	−12.06	17.20
ln(*FC*)	Financing constraints FC index	33,468	0.462	0.278	0	0.988
ln(*AI*)	Degree of artificial intelligence adoption	33,468	11.20	1.686	5.483	14.86
ln(*SIZE*)	Enterprise size	33,468	3.096	0.061	2.383	3.355
*LEV*	Financial leverage	33,468	0.478	1.469	−0.195	142.718
*EAR*	Enterprise profitability	33,468	0.087	0.207	−1.821	3.891
*GRO*	Enterprise growth capacity	33,468	5.088	74.078	−2.733	1,346.071
ln(*YEAR*)	Years of enterprise listed	33,468	2.898	0.451	1.609	3.555
*ROA*	Return on assets	33,468	0.008	1.286	−0.513	235.098

It can be seen from the table that for the explained variable ln(*DIS*), the minimum value is 0.003, the maximum value is 23.719, the variance is 3.403, and the average value is 5.368. This indicates that there is a large gap in the degree of pollution transfer among the sample enterprises selected in this study, and the average degree of pollution transfer is relatively high. For the explanatory variable ln(CPU), the minimum value is −2.308, the maximum value is 0.591, and the variance is 0.959. This shows that the gap in the climate policy uncertainty of the regions where the sample enterprises in this study are located is relatively low, which is in line with the policy stability of the Chinese government. Among the mediating variables of financing constraint SA, WW, KZ, and FC index, the variances of the WW and KZ of the sample enterprises are 25.92 and 2.203, respectively, with a large difference. It indicates that there is a large gap in the earnings before interest and taxes and operating cash flows of the sample enterprises. The minimum value of the moderating variable ln(AI) is 5.483, the maximum value is 14.86, and the variance is 1.686. This shows that the degree of artificial intelligence adoption of the sample enterprises selected in this study is relatively high.

### 4.2 Regression test

This study measures the impact of climate policy uncertainty (ln(*CPU*)) on enterprises' pollution transfer distance (ln(*DIS*)) from three perspectives: the economic center of gravity (ln(*E*_*D*_*IS*)), the environmental regulation center of gravity (ln(*R*_*D*_*IS*)), and the pollution center of gravity (ln(*P*_*D*_*IS*)), by constructing a fixed effects model. The results are shown in the following Table 3:

**Table 3:** The results regression test.

**Variables**	**ln(*DIS*)**	**ln(*E_DIS*)**	**ln(*P_DIS*)**	**ln(*R_DIS*)**	**ln(*DIS*)**
	**(1)**	**(2)**	**(3)**	**(4)**	**(5)**
ln(*CPU*)	0.251^***^ (6.487)	2.428^**^ (156.018)	3.355^**^ (41.749)	0.841^**^ (0.178)	0.249^**^ (6.442)
ln(*SIZE*)	2.227^**^ (4.496)	0.179^**^ (36.778)	0.034 (1.335)	0.018 (0.034)	2.215^**^ (4.343)
*LEV*	−0.096 (−1.799)	−0.014^**^ (−26.862)	0.003 (0.955)	−0.018 (−1.961)	−0.085 (−1.551)
*EAR*	−0.218 (−1.297)	−0.006^**^ (−3.967)	−0.008 (−1.060)	−0.080 (−0.579)	−0.224 (−1.330)
*GRO*	−0.002 (−0.094)	−0.002^**^ (−9.172)	−0.003^**^ (−2.706)	0.060^**^ (2.641)	0.004 (0.190)
ln(*YEAR*)	−0.113^*^ (−1.986)	−0.033^**^ (−57.891)	−0.003 (−1.128)	−0.110^*^ (−2.127)	0.024 (0.214)
*ROA*	−0.083^**^ (−2.648)	−0.010^**^ (−3.308)	0.005^**^ (2.994)	−0.096^**^ (−3.387)	−0.080^*^ (−2.549)
Cons	−12.167^**^ (−7.680)	0.001^**^ (4.205)	−0.002 (−0.873)	−11.347^**^ (−7.761)	−12.493^**^ (−7.641)
Time control	Yes	Yes	Yes	Yes	Yes
City control	Yes	Yes	Yes	Yes	Yes
Enterprises' control	No	No	No	No	Yes
*N*	22,260	22,260	22,260	22,260	22,260
Adj *R*^2^	0.150	0.430	0.432	0.495	0.203

^*^, ^**^ and ^***^ indicate significance at 10%, 5% and 1% level, respectively.

In this study, first, Model (1) analyzes the impact of ln(*CPU*) on ln(*DIS*). The coefficient of ln(*CPU*) on ln(*DIS*) is 0.251. The results show that climate policy uncertainty significantly exacerbates enterprises' pollution transfer behavior, and the degree of enterprises' pollution transfer increases by 0.251 units. As shown in Model (5) in the table, after adding the additional control for individual effects, the estimated value of the coefficient of the impact of climate policy uncertainty on enterprises' pollution transfer behavior is 0.249. Although this effect is somewhat weakened compared with the benchmark model that only controls for time and city fixed effects, it still maintains a positive statistical significance at the 5% significance level (β = 0.249, *P* < 0.05), which is consistent with the benchmark estimation results before adding the enterprises' control effects. These findings further verify the accuracy of the empirical results.

Second, this study analyzes the specific impacts of the climate policy uncertainty of local governments on enterprises' pollution transfer behavior from three perspectives: the location of enterprises' economic centers of gravity, the location of environmental protection investments, and the location of pollution centers of gravity. Models (2), (3), and (4) represent the impacts of climate policy uncertainty on the locations of enterprises' economic centers of gravity, pollution centers of gravity, and environmental regulation centers of gravity, respectively. The coefficient of ln(*CPU*) on ln(*E*_*D*_*IS*), ln(*P*_*D*_*IS*) and ln(*R*_*D*_*IS*) are 2.428, 3.355, and 0.841, respectively. The results of Models (2), (3), and (4) show that as the uncertainty of the climate policies increases, the economic development centers of gravity, environmental protection investment centers of gravity, and pollution behavior centers of gravity of enterprises all shift outward, increasing by 2.428, 3.355, and 0.841 units, respectively. It can be seen that climate policy uncertainty has the greatest impact on the location of local enterprises' pollution emission centers of gravity, followed by the environmental regulation centers of gravity and economic development centers of gravity of enterprises. This indicates that when the climate policy uncertainty is high, enterprises do not reduce pollution emissions but instead transfer pollution emissions to surrounding areas and cities with looser supervision. This is consistent with the research results analyzed earlier, that is, the uncertainty of climate policies will exacerbate enterprises' pollution transfer behavior.

### 4.3 Robustness test

This study conducted robustness regressions by replacing the dependent variable to validate the robustness of the main regression results.

This study adopted different calculation methods and replaced the original explained variable, the enterprise pollution distance (ln(*DIS*)), with the waste gas emission distance (ln(*AIR*)), wastewater emission distance (ln(*Water*)), and waste emission distance, respectively (ln(*Solid*)), and conducted regression tests to verify the robustness of the research results. Moreover, considering that an enterprise's pollution behavior may be affected by other enterprises in the supply chain, this study took the pollution distance of the enterprise's upstream supply enterprises as the explained variable for regression (ln(*SUPPLY*)), so as to verify the robustness of the results.

As can be seen in the Table 4, first, ln(*CPU*) still has a relatively significant positive impact on ln(*AIR*), ln(*Water*) and ln(*Solid*). The coefficients of ln(*CPU*) on ln(*AIR*), ln(*Water*) and ln(*Solid*) are 14.533, 16.734, and 14.592, respectively. This indicates that the higher the climate policy uncertainty, the more severe the waste gas, wastewater, and waste pollution transfer behaviors of enterprises. Second, for upstream supply enterprises, the coefficient is 9.366. This shows that the uncertainty of climate policy will also exacerbate the pollution transfer behaviors of upstream suppliers. This indirectly demonstrates the positive impact relationship between climate policy uncertainty and enterprises' pollution transfer behaviors. Therefore, it can be seen from the results that the findings of this study are somewhat robust.

**Table 4:** The results of robustness test by changed explained variables.

**Variables**	**ln(*SUPPLY*)**	**ln(*AIR*)**	**ln(*Water*)**	**ln(*Solid*)**
	**(1)**	**(2)**	**(3)**	**(4)**
ln(*CPU*)	9.366^*^ (4.553)	14.533^**^ (4.428)	16.734^***^ (4.860)	14.952^**^ (4.478)
ln(*SIZE*)	1.851 (0.968)	1.137 (0.947)	1.664 (0.934)	1.079 (0.948)
*LEV*	−0.203 (6.758)	−1.041 (6.752)	−0.428 (6.729)	−1.074 (0.752)
*EAR*	3.941^***^ (1.090)	2.986^**^ (1.105)	3.275^**^ (1.077)	−9.608 (31.32)
*GRO*	26.260 (23.69)	−10.240 (31.53)	22.450 (22.87)	2.977^**^ (1.103)
ln(*YEAR*)	−0.905 (8.080)	−34.782 (26.38)	−58.854^*^ (25.04)	−34.372 (26.34)
*ROA*	0.0166 (0.0367)	3.845^**^ (1.167)	3.941^***^ (1.090)	2.977^**^ (1.103)
Cons	−62.640^*^ (25.97)	−34.784 (26.38)	−58.851^*^ (25.04)	1.079 (0.948)
Time control	Yes	Yes	Yes	Yes
City control	Yes	Yes	Yes	Yes
Id control	Yes	Yes	Yes	Yes
*N*	33,468	33,468	33,468	33,468
Adj *R*^2^	0.224	0.469	0.383	0.009

^*^, ^**^ and ^***^ indicate significance at 10%, 5% and 1% level, respectively.

### 4.4 Endogeneity tests

In this study, the original fixed effects model was replaced with the GMM model, respectively, and the climate policy uncertainty with a one period lag (ln(*L*.*CPU*)) was used as an instrumental variable to conduct endogeneity tests separately, so as to verify the endogeneity of the research results.

As can be seen from the results in the Table 5, first, in Model (1), AR(1) is 0.04, AR(2) is 0.614, and the Sargan test value is 0.812. This indicates that the GMM model can effectively identify the endogeneity problems in the model established in this study. In the GMM model, climate policy uncertainty still has a relatively significant positive promoting effect on enterprises' pollution transfer behavior, suggesting that the model established in this study does not have serious endogeneity problems. Secondly, in Model (2), the Wald *F* statistic is 79.364, indicating that the regression model of ln(*CPU*) on ln(*DIS*) does not have serious endogeneity problems. Therefore, the instrumental variables selected in this study are relatively appropriate. Finally, the instrumental variables still have a positive impact on enterprises' pollution transfer behavior, verifying that the research results in the previous text do not have serious endogeneity problems.

**Table 5:** The results of endogeneity test.

**Variables**	**ln(*DIS*)**	**ln(*DIS*)**
	**(1)**	**(2)**
ln(*L*.*CPU*)		0.251^**^ (6.116)
ln(*CPU*)	0.114^*^ (2.441)	
ln(*SIZE*)	0.818 (0.728)	2.227^**^ (4.399)
*LEV*	0.107 (0.850)	−0.096 (−1.835)
*EAR*	−0.052 (−1.092)	−0.002 (−0.091)
*GRO*	−0.047 (−0.987)	−0.218 (−1.454)
ln(*YEAR*)	0.005 (0.046)	−0.113^*^ (−2.019)
*ROA*	−0.024 (−0.459)	−0.083^**^ (−2.683)
Cons	−0.047 (−0.987)	−12.167^**^ (−7.516)
AR(1)	0.004	
AR(2)	0.614	
Sargan test	0.812	
Kleibergen-Paap rk Wald *F* statistic		79.364
Chi-sq(1) *P*-value		0.0000

^*^ and ^**^ indicate significance at 10% and 5% level, respectively.

### 4.5 Mechanism test

#### 4.5.1 Mediating mechanism test

As can be seen from the above results of the main effect test, the uncertainty of climate policies will exacerbate enterprises' pollution transfer behavior. With the increase in climate policy uncertainty, the external risks faced by enterprises will rise. The financial institutions will then raise their assessment of the expected future risks of enterprises, thus increasing the financing thresholds and costs for enterprises. Therefore, when faced with the choice between green innovation and pollution transfer, enterprises are more inclined to shift their economic focus, environmental regulation focus, and pollution focus to surrounding areas, thereby reducing the impact of climate policy uncertainty on themselves. This study used the FC, SA, WW, and KZ, respectively to measure the financing constraints of enterprises, and the results are shown in the Table 6.

**Table 6:** The results of mediating mechanism test.

**Variables**	**ln(*FC*)**	**ln(*KZ*)**	**ln(*WW*)**	**ln(*SA*)**
	**(1)**	**(2)**	**(3)**	**(4)**
ln(*CPU*)	0.022^**^ (2.974)	0.385^**^ (3.421)	0.013^**^ (3.093)	0.139^**^ (30.571)
ln(*SIZE*)	−0.417^**^ (−2.873)	−11.723^**^ (−21.656)	−1.524^**^ (−28.909)	−3.102^**^ (−52.823)
*LEV*	−0.003 (−0.412)	2.232^**^ (40.666)	−0.116^**^ (−19.899)	−0.144^**^ (−28.827)
*EAR*	0.040^**^ (20.335)	−0.011 (−0.903)	0.001 (1.784)	0.007^**^ (3.978)
*GRO*	−0.001^*^ (−2.157)	0.006 (0.933)	0.018 (1.119)	0.016 (0.755)
ln(*YEAR*)	0.076^**^ (23.330)	0.023 (1.258)	−0.116^**^ (−19.899)	−0.003 (−1.338)
*ROA*	0.034^**^ (5.366)	−0.340 (−1.418)	0.015^**^ (2.872)	0.018 (0.788)
Cons	−2.694^**^ (−5.946)	31.890^**^ (22.282)	3.513^**^ (20.726)	9.939^**^ (52.580)
Time control	Yes	Yes	Yes	Yes
City control	Yes	Yes	Yes	Yes
Id control	Yes	Yes	Yes	Yes
*N*	33,468	33,468	33,468	33,468
Adj *R*^2^	0.188	0.551	0.547	0.308

^*^ and ^**^ indicate significance at 10% and 5% level, respectively.

First, this study uses the ln(*FC*) to measure the financing constraints of enterprises. The coefficient of ln(*CPU*) in Model (1) is 0.022, which indicates that climate policy uncertainty increases the ln(*FC*), meaning that climate policy uncertainty raises the financing constraints on enterprises. However, the ln(*FC*) is calculated through standardization based on indicators such as enterprise size and age. Therefore, the ln(*FC*) may have strong endogeneity. The KZ index, WW index, and SA index can better control the endogeneity problem. Among them, the SA index performs the best in endogeneity control, followed by the WW index and the KZ index. Therefore, according to the effect of endogeneity control, this study successively uses the ln(*KZ*), ln(*WW*), and ln(*SA*) index to replace the FC index for regression tests. The coefficients of ln(*CPU*) on ln(*KZ*), ln(*WW*), and ln(*SA*) are 0.385, 0.013, and 0.139, respectively. The results show that climate policy uncertainty has a significant positive impact on the KZ, WW, and SA index, that is, climate policy uncertainty increases the financing constraints of enterprises.

On the one hand, from the perspective of cost benefit analysis, financing constraints increase the pressure of compliance costs on enterprises. In order to meet emission standards, whether it is to replace with cleaner production equipment or to carry out green environmental protection technology innovation, the demand for funds is huge. Therefore, the pressure of financing constraints forces enterprises that are unable to bear the environmental protection costs to choose to transfer pollution to neighboring cities with relatively loose environmental supervision, so as to avoid high pollution treatment costs. Through pollution transfer behavior, enterprises can quickly ease their financial difficulties, concentrate limited funds on core profit-making businesses, and sacrifice the environment for living space. On the other hand, the difference in economic development levels among regions further amplifies the pollution transfer behavior of enterprises. Economically developed regions have more diversified financing channels, while enterprises in underdeveloped regions face greater difficulties in financing. To undertake industrial transfer and promote local employment and economic growth, underdeveloped regions often relax the threshold of environmental regulation. This forms a push pull mechanism. Enterprises affected by financing constraints are squeezed out from developed regions and attracted by underdeveloped regions, accelerating cross regional pollution transfer, disrupting the ecological balance, and exacerbating regional environmental inequality.

Therefore, this study verifies Hypothesis 2. The uncertainty of climate policy exacerbates the pollution transfer behavior of enterprises through financing constraints.

#### 4.5.2 Moderating effect test

In this study, by introducing the interaction term ln(*CCPU* × *AI*) of climate policy uncertainty ln(*CCPU*) and the degree of artificial intelligence adoption ln(*AI*), regression analyses were, respectively conducted on the enterprise pollution transfer behavior ln(*DIS*) and the financing constraint indices such as SA, KZ, FC, and WW. The results are shown in the following Table 7:

**Table 7:** The results of moderating effect test.

**Variables**	**ln(*DIS*)**	**SA**	**KZ**	**FC**	**WW**
	**(1)**	**(2)**	**(3)**	**(4)**	**(5)**
ln(*AI*)	−0.218^*^ (−2.331)	−0.001^*^ (−2.198)	−0.072^**^ (−3.454)	−0.012^**^ (−5.396)	−0.067^**^ (−6.001)
ln(*CPU*)	−0.504^**^ (−3.156)	−0.024^**^ (−3.108)	−0.373^**^ (−2.993)	−0.148^**^ (−20.922)	0.014^**^ (2.986)
ln(*CPU* × *AI*)	−0.320^*^ (−2.125)	−0.049^**^ (−21.342)	−2.234^**^ (−36.505)	−0.005^*^ (−2.450)	−0.014^**^ (−2.986)
ln(*SIZE*)	−1.548 (−0.856)	−0.425^**^ (−2.830)	−9.149^**^ (−20.482)	−3.079^**^ (−52.836)	−1.464^**^ (−23.617)
*LEV*	0.094 (0.503)	−0.042^**^ (−17.597)	−2.536^**^ (−48.211)	−0.145^**^ (−28.439)	−0.119^**^ (−17.879)
*EAR*	0.034 (0.342)	−0.012 (−1.311)	0.026 (1.210)	0.003 (0.942)	0.019 (1.115)
*GRO*	0.006 (0.611)	0.031 (1.476)	0.026 (1.210)	−0.015 (−1.202)	0.002 (1.715)
ln(*YEAR*)	0.123 (1.409)	0.080^**^ (20.972)	2.536^**^ (48.211)	−0.004 (−1.626)	0.071^**^ (14.446)
*ROA*	−0.109 (−1.616)	0.004 (1.374)	−0.616 (−1.543)	0.009 (0.421)	0.010 (1.407)
Cons	9.582 (1.595)	−2.678^**^ (−5.689)	32.065^**^ (22.465)	9.871^**^ (52.681)	3.320^**^ (16.652)
Time control	Yes	Yes	Yes	Yes	Yes
City control	Yes	Yes	Yes	Yes	Yes
Id control	Yes	Yes	Yes	Yes	Yes
*N*	33,468	33,468	33,468	33,468	33,468
Adj *R*^2^	0.115	0.551	0.450	0.308	0.994

As can be seen in the Table 7, the coefficient of the degree of artificial intelligence adoption ln(*AI*) on enterprise pollution transfer ln(*DIS*) is −0.218 in model (1), indicating that the degree of artificial intelligence adoption can inhibit the enterprise pollution transfer behavior. The coefficient of ln(*CPU* × *AI*) on ln(*DIS*) is 0.320, suggesting that compared with enterprises with a low degree of artificial intelligence adoption, when external climate policies change, enterprises with a higher degree of artificial intelligence adoption face relatively lower expected risks in the future. This makes such enterprises less likely to transfer pollution to surrounding cities with stable policies compared to those with a lower degree of artificial intelligence adoption when dealing with policy changes. Moreover, the regression coefficients of the degree of ln(*AI*) on the enterprise financing constraint indices SA, KZ, FC, and WW are −0.01, −0.072, −0.012, and −0.067, respectively. This shows that the degree of artificial intelligence adoption can effectively alleviate the financing constraint problems faced by enterprises. And the regression coefficients of the interaction term ln(*CCPU* × *AI*) on financing constraints are −0.049, −2.234, −0.05, and −0.014, respectively, indicating that the degree of artificial intelligence adoption can effectively mitigate the negative impact of climate policy uncertainty on enterprise financing constraints.

### 4.6 Heterogeneity analysis

#### 4.6.1 Industry analysis

In this study, based on the classification of polluting and non-polluting enterprises by the Chinese government, 16 polluting industries with industry codes such as B06, C17, D44, etc. are defined as heavily polluting enterprises. These include industries such as the coal industry, textile industry, and power industry. Other enterprises are defined as non-heavily polluting enterprises. Eventually, this study screened out 7,910 observations of heavily polluting enterprises and 25,558 observations of non-heavily polluting enterprises, and conducted regression analyses, respectively. The pollution transfer distance of the two types of enterprises is defined as ln(*H*_*D*_*IS*) and ln(*NH*_*D*_*IS*), respectively. The results are as follows:

As can be seen from the Table 8, the coefficient of ln(*CPU*) on ln(*NH*_*D*_*IS*) is 0.128, while that the coefficient of ln(*CPU*) on ln(*H*_*D*_*IS*) is 2.652. It indicates that ln(*CPU*) has a greater impact on ln(*H*_*D*_*IS*) than ln(*NH*_*D*_*IS*). Among them, heavily polluting industries such as energy, printing, and metal are more significantly affected. This is mainly because these heavily polluting enterprises need to invest relatively high costs when carrying out green technology innovation or fulfilling social responsibilities, thus making the costs of their green transformation remain high. Moreover, the green transformation of heavily polluting enterprises is generally accompanied by problems such as high risks and long investment payback periods. Therefore, when such enterprises face a highly uncertain external policy environment, among the two coping options of green technology innovation and pollution transfer, they tend to transfer funds and resources to surrounding cities with more stable policies to avoid policy risks. In contrast, non-heavily polluting enterprises face relatively low emission reduction costs and have little difficulty in achieving the emission reduction targets set by the government. Based on cost benefit considerations, when facing climate policy uncertainty, the willingness of non-heavily polluting enterprises to transfer pollution is far less strong than that of heavily polluting enterprises. Therefore, when the uncertainty of climate policies is high, heavily polluting enterprises face higher policy risks.

**Table 8:** The results of industry heterogeneity analysis.

**Variables**	**ln(*NH_DIS*)**	**ln(*H_DIS*)**
	**(1)**	**(2)**
ln(*CPU*)	0.128^**^ (63.538)	2.652^**^ (2.068)
ln(*SIZE*)	−0.160 (−1.210)	−0.005 (−0.046)
*LEV*	−0.080 (−1.489)	−0.001 (−0.059)
*EAR*	0.004^**^ (4.616)	−0.246 (−0.640)
*GRO*	−0.002 (−0.232)	0.006 (0.866)
ln(*YEAR*)	0.589 (1.904)	0.023 (0.199)
*ROA*	0.006 (0.866)	0.006 (0.115)
Cons	3.188 (1.242)	1.314 (0.510)
Time control	Yes	Yes
City control	Yes	Yes
Id control	Yes	Yes
*N*	7,910	25,558
Adj *R*^2^	0.313	0.276

^**^ indicate significance 5% level.

#### 4.6.2 Ownership analysis

In this study, the sample enterprises are classified into three categories according to the shares held by the central government or local governments and non-state owned enterprises, namely *CSOEs*, *LSOEs*, and *NSOEs*. Eventually, this study has screened out 1,657 observations for *CSOEs*, 11,979 observations for *LSOEs*, and 19,832 observations for *NSOEs*. The pollution transfer distance of the different ownership types of enterprises is defined as ln(*CSOEs*_*D*_*IS*), ln(*LSOEs*_*D*_*IS*), and ln(*NSOEs*_*D*_*IS*), respectively. The regression analyses are conducted, respectively, and the results are as follows:

As can be seen in the Table 9, the coefficients of climate policy uncertainty ln(*CPU*) on the pollution transfer behavior of central SOEs, local SOEs, and NSOEs are 0.029, 0.408, and 1.024, respectively. This indicates that compared with central SOEs and local SOEs, climate policy uncertainty has a greater impact on NSOEs, followed by central SOEs, and finally, climate policy uncertainty has the least impact on local SOEs. The main reason might be that, compared with central SOEs and local SOEs, NSOEs have fewer financing channels and rely more on financing channels of third party financial institutions such as commercial banks. Therefore, the uncertainty of climate policies undoubtedly increases the expected future risks of these NSOEs. In response, the financial institutions such as commercial banks have raised the financing thresholds and costs for these enterprises. For central SOEs and local SOEs, they have the national or local government as a background resource, so third party financial institutions such as commercial banks have a lower assessment of their expected future risks. Consequently, climate policy uncertainty has a greater impact on NSOEs.

**Table 9:** The results of industry ownership analysis.

**Variables**	**ln(*CSOEs_DIS*)**	**ln(*LSOEs_DIS*)**	**ln(*NSOEs_DIS*)**
	**(1)**	**(2)**	**(3)**
ln(*CPU*)	0.029^**^ (18.244)	0.408^*^ (−2.317)	1.024^***^ (1.964)
ln(*SIZE*)	0.067^**^ (13.209)	2.735 (1.373)	1.152 (1.527)
*LEV*	0.116^**^ (36.748)	−0.083 (−0.292)	0.030^*^ (1.990)
*EAR*	−0.202^**^ (−20.530)	−0.266 (−0.488)	−0.076 (−0.115)
*GRO*	−0.085^**^ (−15.017)	0.013 (0.266)	0.083 (0.462)
ln(*YEAR*)	0.150^**^ (59.281)	0.047 (0.465)	1.463 (1.803)
*ROA*	0.111^**^ (36.144)	0.047 (0.465)	−0.166 (−0.641)
Cons	0.067^**^ (13.718)	−12.516 (−1.958)	−6.552 (−1.727)
Time control	Yes	Yes	Yes
City control	Yes	Yes	Yes
Id control	Yes	Yes	Yes
*N*	1,657	11,979	19,832
Adj *R*^2^	0.264	0.544	0.481

^*^, ^**^ and ^***^ indicate significance at 10%, 5% and 1% level, respectively.

## 5 Conclusion

This study focuses on the impact of climate policy uncertainty on enterprises' “pollution migration” in the context of artificial intelligence. Through theoretical analysis and empirical testing, the following key conclusions are drawn:

Firstly, climate policy uncertainty significantly exacerbates enterprises' pollution migration behavior. When climate policy uncertainty increases, government-related uncertainties rise, external risks for enterprises increase, it becomes difficult for enterprises to accurately predict environmental protection costs, the market competition pattern is also affected, and the motivation for green technology innovation weakens. To reduce environmental cost risks and gain a competitive advantage, enterprises tend to transfer pollution intensive production processes to surrounding areas with lenient climate policies and weak supervision, thus leading to an increase in pollution migration behavior.

Secondly, climate policy uncertainty increases enterprises' financing constraints, and higher financing constraints, in turn, exacerbate enterprises' pollution transfer. Climate policy uncertainty increases enterprises' expected future operating risks and costs. Third party financial institutions such as commercial banks and securities institutions will accordingly raise their assessment of enterprises' expected risks, and then increase the financing threshold and costs for enterprises, putting enterprises in a financing predicament. Since the costs of emission reduction and green technology innovation are high, the returns are low, and the payback period is long, the increase in financing constraints restricts enterprises' investment in these aspects. This prompts enterprises to choose pollution transfer to relieve short term environmental cost pressure and maintain their survival and competitive position.

Thirdly, the degree of artificial intelligence adoption plays a certain moderating role in the impact of climate policy uncertainty on enterprises' financing constraints. On the one hand, compared with enterprises with a low degree of artificial intelligence adoption, enterprises with a high degree of artificial intelligence adoption have higher-quality information disclosure and greater transparency. This can reduce information asymmetry, enabling them to obtain more favorable financing conditions and ease financing constraints. On the other hand, such enterprises have higher investment efficiency and stronger green innovation capabilities. When facing financing constraints, they can still maintain a certain pollution control capacity and reduce the motivation for pollution transfer. Therefore, enterprises with a high degree of artificial intelligence adoption are less affected by climate policy uncertainty in terms of pollution transfer behavior.

Finally, on the one hand, from the perspective of industry nature, compared with non-heavily polluting enterprises, the impact of climate policy uncertainty on the pollution transfer behavior of heavily polluting enterprises is more significant. Heavily polluting enterprises have high pollution emissions during the production process, face stricter environmental supervision and higher emission reduction costs. Policy uncertainty makes it difficult for them to predict compliance costs, greatly increasing their operating risks and cost expectations, thus making pollution transfer more likely to occur. On the other hand, from the perspective of enterprise attributes, compared with SOEs, climate policy uncertainty has a greater impact on the pollution transfer behavior of NSOEs. The NSOEs are at a relative disadvantage in terms of resource acquisition, policy support, and financing channels. They face higher financing constraint thresholds and costs, have low flexibility in dealing with policy risks, and are more inclined to reduce short-term costs and risks through pollution transfer.

### 5.1 Discussion

Climate change is not only a major challenge that humanity urgently needs to face, but also has a profound impact on the stable development of the global economy. The issue of climate policy uncertainty arising from climate change responses has become a key factor influencing enterprises' investment decisions, forcing enterprises to consider the potential risks brought about by policy changes when formulating strategies (77). At the same time, the degree of artificial intelligence adoption, as a key transformation in the development mode of enterprises, plays a crucial role in alleviating the negative impacts of information asymmetry on the operation process of enterprises. Therefore, this study selects Chinese listed companies from 2010 to 2022 to explore the impact and action mechanism of climate policy uncertainty on enterprises' “pollution migration” behavior, and further examines the moderating effect of the degree of enterprises' artificial intelligence adoption. The results show that: firstly, climate policy uncertainty significantly exacerbates enterprises' “pollution migration” behavior. Secondly, the degree of artificial intelligence adoption can significantly resist the exacerbating effect of climate policy uncertainty on “pollution migration.” Thirdly, financing constraints are an important transmission channel through which climate policy uncertainty affects enterprises' “pollution migration” behavior. Fourthly, the impact of climate policy uncertainty on enterprises' “pollution migration” behavior is more significant in NSOEs and heavily polluting enterprises. Therefore, this study draws the following policy implications:

First, this study verifies that climate policy uncertainty exacerbates enterprises' pollution transfer behavior. This conclusion is consistent with the “pollution haven” hypothesis, indicating that when facing policy risks, enterprises will seek regions with more stable regulatory policies to reduce environmental costs. Financing constraints play a certain mediating role, further revealing the economic logic behind enterprise decisions, that is, how financial factors affect enterprises' strategies in response to environmental policies. In addition, the moderating effect of the degree of artificial intelligence adoption reflects the importance of artificial intelligence technology in enterprises' environmental decisions, providing a new perspective for studying the relationship between enterprises' environmental behavior and technological innovation.

Second, from the perspective of policy making, the government needs to recognize the crucial role of stable climate policies in guiding enterprises toward green development. Frequent policy changes will lead enterprises to engage in short-term behaviors and exacerbate pollution transfer. Therefore, the government should enhance the forward-looking and coherence of policies to reduce uncertainty. For example, when formulating carbon tax policies, clarify the implementation details and adjustment mechanisms in advance so that enterprises can reasonably plan their environmental protection investments (79). In terms of financial support, it is necessary to improve the green financial system. According to the characteristics of different types of enterprises, develop a variety of financial products to reduce the financing costs of heavily polluting enterprises and NSOEs and encourage them to carry out green transformation. At the same time, increase support for enterprises' application of artificial intelligence. Through subsidies, tax preferences, and other means, raise the degree of artificial intelligence adoption by enterprises and enhance their ability to respond to changes in environmental policies. For enterprises, they should actively pay attention to the dynamics of climate policies, take the initiative to improve the level of artificial intelligence application, and regard it as an important means to enhance competitiveness and respond to environmental risks. By optimizing production processes and strengthening green innovation, reduce their reliance on pollution transfer and achieve sustainable development (78).

### 5.2 Limitation

First, in terms of sample selection, this study only selected listed enterprises in 31 provinces of China from 2010 to 2022 as samples. The sample scope and time span are relatively narrow. Future research can be extended to enterprises in different countries and regions, covering more industries and enterprise types.

Second, the conclusions of this study may be influenced by China's unique institutional environment, with certain limitations. From the perspective of local governments, local governments in China have strong autonomous decision-making power in economic development and environmental supervision, and there exists a “GDP tournament”- style competition logic. This strong influence may further amplify the uncertainty of climate policies. Local governments may relax environmental supervision to attract investment, forming a “race to the bottom,” which makes it easier for enterprises to achieve pollution migration through cross-regional relocation. In many Western federal countries, although local governments have certain autonomy, the policy coordination mechanism between the central and local governments is more mature, and environmental standards are relatively unified. The space for local governments to intervene in enterprise pollution behaviors is smaller, so the driving effect of climate policy uncertainty on pollution migration may be weaker than that in China.

Third, in terms of differences in enterprise ownership, there is a significant imbalance between state-owned enterprises (SOEs) and non-state-owned enterprises (NSOEs) in China in terms of resource acquisition and policy support. SOEs, backed by government credit, can more easily obtain financing support and have stronger capabilities to cope with policy risks. In contrast, NSOEs face restricted financing channels and are more inclined to alleviate pressures through pollution migration. However, in countries with mature market economies, the impact of differences in enterprise ownership on financing and policy responses is weaker. Governments rarely intervene directly in enterprises, and enterprise decisions rely more on market mechanisms. Therefore, the moderating effect of “ownership heterogeneity” on pollution migration may be less obvious.

Fourth, to further enhance the credibility of causal inference regarding climate policy uncertainty (CPU), this study incorporates exogenous policy shocks into the analytical framework to identify their driving effects on CPU. Global climate governance events such as the signing of the Paris Agreement (2015) and successive United Nations Climate Change Conferences (COP summits) constitute typical exogenous shocks. These events directly influence market expectations regarding the direction of climate policies in various countries by setting global emission reduction targets, promoting international policy coordination, or exposing differences in international negotiations. For instance, after the signing of the Paris Agreement, the differences in the pace and intensity of policy adjustments among countries in implementing their Nationally Determined Contributions (NDCs), as well as the outcomes of negotiations on issues such as emission reduction responsibilities and funding mechanisms during COP summits, may all exacerbate enterprises' perceived uncertainty about future policy details such as climate regulatory standards and carbon pricing mechanisms. Analyzing such exogenous events as instrumental variables or shock sources for CPU can effectively eliminate endogeneity interference, more accurately identify the causal impact of climate policy uncertainty on enterprises' pollution migration behavior, and thus strengthen the robustness of the research conclusions.

Finally, China's green financial system is still in the stage of improvement, and the financing support for enterprises' green transformation is insufficient, which exacerbates the mediating effect of financing constraints on pollution migration. In countries with developed green financial markets, enterprises have smoother access to financing instruments such as green credit and green bonds, so the impact of financing constraints may be weakened, and the logic that climate policy uncertainty drives pollution migration through financing constraints will also be correspondingly weakened. Therefore, the conclusions of this study may be biased in countries with strong policy uniformity, restricted local government powers, insignificant ownership differences, or mature financial markets. In subsequent research, in-depth analysis will be conducted in combination with these limitations.

## Data Availability

The original contributions presented in the study are included in the article/Supplementary material, further inquiries can be directed to the corresponding author.

## References

[1] Bergquist P Mildenberger M Stokes LC. Combining climate, economic, and social policy builds public support for climate action in the US. Environ Res Lett. (2020) 15:054019. 10.1088/1748-9326/ab81c1

[2] Stechemesser A Koch N Mark E Dilger E Klösel P Menicacci L. Climate policies that achieved major emission reductions: Global evidence from two decades. Science. (2024) 385:884–92. 10.1126/science.adl6547 39172830

[3] Ren X Li W Duan K Zhang X. Does climate policy uncertainty really affect corporate financialization? Environ Dev Sustain. (2023). 10.2139/ssrn.4079217

[4] Meng J Lu F Cheng B. China's climate change policy attention and forestry carbon sequestration growth. Forests. (2023) 14:2273. 10.3390/f14112273

[5] Zhang G Wang J Liu Y. Energy transition in China: is there a role for climate policy uncertainty? J Environ Manage. (2024) 370:122814. 10.1016/j.jenvman.2024.122814 39378810

[6] Li B Fu E Yang S Lin J Zhang W Zhang J. Measuring China's policy stringency on climate change for 1954–2022. Sci Data. (2025) 12:188. 10.1038/s41597-025-04476-0 39890831 PMC11785789

[7] Arouri M Gomes M Pijourlet G. Does climate policy uncertainty shape the response of stock markets to oil price changes? Evidence from GCC stock markets. J Environ Manage. (2025) 375:124229. 10.1016/j.jenvman.2025.124229 39842356

[8] Gu K Dong F Sun H Zhou Y. How economic policy uncertainty processes impact on inclusive green growth in emerging industrialized countries: a case study of China. J Clean Prod. (2021) 322:128963. 10.1016/j.jclepro.2021.128963

[9] Luers A Koomey J Masanet E Gaffney O Creutzig F Lavista Ferres J. Will AI accelerate or delay the race to net-zero emissions? Nature. (2024) 628:718–20. 10.1038/d41586-024-01137-x 38649764

[10] Sun J Guan X Zeng Y Zhang J Chen X Zhan X. Addressing pollution challenges for enterprises under diverse extreme climate conditions: artificial intelligence-driven experience and policy support of top Chinese enterprises. Front Public Health. (2024) 12:1436304. 10.3389/fpubh.2024.1436304 39301513 PMC11410622

[11] Li H Tian H Zhou W Wu YJ. The impact of artificial intelligence adoption degree on corporate digital technology innovation. Enterprise Information Systems. (2025)

[12] Agrawal R Priyadarshinee P Kumar A Luthra S Garza-Reyes JA Kadyan S. Are emerging technologies unlocking the potential of sustainable practices in the context of a net-zero economy? An analysis of driving forces. Environm Sci Pollut Res. (2025) 32:7130–48. 10.1007/s11356-023-26434-2 36934193 PMC10024615

[13] Kong Q Li R Wang Z Peng D. Economic policy uncertainty and firm investment decisions: dilemma or opportunity? Int Rev Financ Anal. (2022) 83:102301. 10.1016/j.irfa.2022.102301

[14] Wang Y Chen CR Huang YS. Economic policy uncertainty and corporate investment: evidence from China. Pac-Basin Financ J. (2014) 26:227–43. 10.1016/j.pacfin.2013.12.008

[15] Gavriilidis K. Measuring climate policy uncertainty. SSRN Electron J. (2021). 10.2139/ssrn.3847388

[16] Ren X Shi Y Jin C. Climate policy uncertainty and corporate investment: evidence from the Chinese energy industry. Carbon Neutral. (2022) 1:1–14. 10.1007/s43979-022-00008-6

[17] Lan Q Ma P Feng S Tan Y Liu S Zhai Y. Air pollution and corporate financial assets allocation: evidence from China. J Clean Prod. (2024) 469:143195. 10.1016/j.jclepro.2024.143195

[18] von Dulong A Hagen A. Institutions make a difference: assessing the predictors of climate policy stringency using machine learning. Environ Res Lett. (2024) 20:14–56. 10.1088/1748-9326/ada0cb

[19] Lu H Hunt A Morley B. The impact of heterogeneous environmental regulation tools on economic growth: can environmental protection and economic growth be win-win? Sustainability. (2024) 16:5585. 10.3390/su1613558537306881

[20] Pata SK. Comparative impacts of energy, climate, and economic policy uncertainties on renewable energy. J Environ Manage. (2024) 370:122494. 10.1016/j.jenvman.2024.122494 39278022

[21] Bellassen V Shishlov I. Pricing monitoring uncertainty in climate policy. Environ Res Econom. (2016) 68:949–74. 10.1007/s10640-016-0055-x

[22] Guo N Jia S Liu Y. Climate policy uncertainty and its impact on the volatility, correlation, and hedging of sovereign and green bonds. J Environ Manage. (2024) 370:122953. 10.1016/j.jenvman.2024.122953 39447369

[23] Guo X Cheng P Choi B. Impact of corporate environmental uncertainty on environmental, social, and governance performance: the role of government, investors, and geopolitical risk. PLoS ONE. (2024) 19:e0309559. 10.1371/journal.pone.0309559 39190725 PMC11349233

[24] Morão H. Uncertainty in climate policy and energy industry. Energy. (2025) 328. 10.1016/j.energy.2025.136013

[25] Harmsen M Tabak C Höglund-Isaksson L Humpenöder F Purohit P van Vuuren D. Uncertainty in non-CO_2_ greenhouse gas mitigation contributes to ambiguity in global climate policy feasibility. Nat Commun. (2023) 14:2949. 10.1038/s41467-023-38577-4 37268633 PMC10238505

[26] Huang W. Climate policy uncertainty and green innovation. Econ Lett. (2023) 233:111423. 10.1016/j.econlet.2023.111423

[27] Bai D Du L Xu Y Abbas S. Climate policy uncertainty and corporate green innovation: evidence from Chinese A-share listed industrial corporations. Energy Econ. (2023) 127:107020. 10.1016/j.eneco.2023.107020

[28] Attílio LA. Spillover effects of climate policy uncertainty on green innovation. J Environ Manage. (2025) 375:124334. 10.1016/j.jenvman.2025.124334 39889428

[29] Lian C Pei J Zheng S Li B. How does trade policy uncertainty affect green innovation in the USA and China? A nonlinear perspective. Environ Sci Pollut Res. (2024) 31:19615–34. 10.1007/s11356-024-31954-6 38363502

[30] Sun G Fang J Li T Ai Y. Effects of climate policy uncertainty on green innovation in Chinese enterprises. International Review of Financial Analysis (Online)/International Review of Financial Analysis (2024). 10.1016/j.irfa.2023.10296039889428

[31] Golub AA Fuss S Lubowski R Hiller J Khabarov N Koch N. Escaping the climate policy uncertainty trap: options contracts for REDD+. Climate Policy. (2018) 18:1227–34. 10.1080/14693062.2017.1422478

[32] Mokni K Hedhili Zaier L Youssef M Ben Jabeur S. Quantile connectedness between the climate policy and economic uncertainty: evidence from the G7 countries. J Environ Manage. (2024) 351:119826. 10.1016/j.jenvman.2023.119826 38147765

[33] Ayed S Ben-Amar W Arouri M. Climate policy uncertainty and corporate dividends. Financ Res Lett. (2024) 60:104948. 10.1016/j.frl.2023.104948

[34] Lu C Ouyang Q. Environmental regulation and urban position in the inter-urban pollution transfer network: a perspective on network analysis of pollution-intensive enterprises' relocation. J Clean Prod. (2023) 435:140418. 10.1016/j.jclepro.2023.140418

[35] Li X Wang R Shen Z Song M. Government environmental signals, government–Enterprise collusion and corporate pollution transfer. Energy Econo. (2024) 139:107935. 10.1016/j.eneco.2024.107935

[36] Zhang C Tao R Yue Z Su F. Regional competition, rural pollution haven and environmental injustice in China. Ecol Econom. (2023) 204:107669. 10.1016/j.ecolecon.2022.107669

[37] Yu X Wan K Du Q. Can carbon market policies achieve a “point-to-surface” effect?—Quasi-experimental evidence from China. Energy Policy. (2023) 183:113803. 10.1016/j.enpol.2023.113803

[38] Meah N. Climate uncertainty and policy making—what do policy makers want to know? Reg Environ Change. (2019) 19:1611–21. 10.1007/s10113-019-01492-w

[39] Lu H Hunt A. Towards sustainability: how does climate policy uncertainty affect regional green innovation in China? Sustainability. (2025) 17:2857. 10.3390/su17072857

[40] Xu X Huang S Lucey BM An H. The impacts of climate policy uncertainty on stock markets: comparison between China and the US. Int Rev Financ Anal. (2023) 88:102671. 10.1016/j.irfa.2023.10267138582094

[41] Kim Y Park YK Ryu D. Climate policy uncertainty and corporate environmental risk-taking. Financ Res Lett. (2025) 82:107555. 10.1016/j.frl.2025.107555

[42] Li M Gao Y Meng B Yang Z. Managing the mitigation: analysis of the effectiveness of target-based policies on China's provincial carbon emission and transfer. Energy Policy. (2021) 151:112189. 10.1016/j.enpol.2021.112189

[43] Sun H Lu H Hunt A. Climate policy uncertainty and corporate green governance: evidence from China. Systems. (2025) 13:635. 10.3390/systems1308063537648195

[44] Gounopoulos D Zhang Y. Temperature trend and corporate cash holdings. Financial Management. (2024) 10.1111/fima.12451

[45] Aglietta M Hourcade J.-C, Jaeger C, Fabert BP. Financing transition in an adverse context: climate finance beyond carbon finance. Int Environ Agreement Polit Law Econom. (2015) 15:403–20. 10.1007/s10784-015-9298-1

[46] Yang J Luo P Tan Y. Contingent decision of corporate environmental responsibility based on uncertain economic policy. Sustainability. (2020) 12:8839. 10.3390/su12218839

[47] An S Li B Song D Chen X. Green credit financing versus trade credit financing in a supply chain with carbon emission limits. Eur J Operat Res. (2020) 292:125–42. 10.1016/j.ejor.2020.10.025

[48] Ghisetti C Mancinelli S Mazzanti M Zoli M. Financial barriers and environmental innovations: evidence from EU manufacturing firms. Climate Policy. (2016) 17:S131–47. 10.1080/14693062.2016.1242057

[49] Lu H Hunt A. Impact of climate policy uncertainty on regional new quality productive forces in China. Urban Sci. (2025) 9:189. 10.3390/urbansci9060189

[50] Narita D Sato I Ogawada D Matsumura A. Integrating economic measures of adaptation effectiveness into climate change interventions: a case study of irrigation development in Mwea, Kenya. PLoS ONE. (2020) 15:e0243779. 10.1371/journal.pone.0243779 33306704 PMC7732349

[51] Chu Z Zhang Z Tan W Chen P. Revolutionizing energy practices: unleashing the power of artificial intelligence in corporate energy transition. J Environ Manage. (2024) 357:120806. 10.1016/j.jenvman.2024.120806 38583377

[52] Yang W Yang G Yang G. The impact of AI on enterprise energy management: from the perspective of carbon emissions. Sci Technol Energy Trans. (2024) 80:8. 10.2516/stet/2024096

[53] Liu X Cifuentes-Faura J Zhao S Wang L Yao J. Impact of artificial intelligence technology applications on corporate energy consumption intensity. Gondwana Res. (2025). 138:89–103. 10.1016/j.gr.2024.09.00337318732

[54] Cheng K Jin Z Wu G. Unveiling the role of artificial intelligence in influencing enterprise environmental performance: evidence from China. J Clea Prod. (2024) 440:140934. 10.1016/j.jclepro.2024.14093439490017

[55] Li S Younas MW Maqsood US Zahid RA. Impact of AI adoption on ESG performance: evidence from Chinese firms. Energy & Environment (2024).38677408

[56] Shao J Lou Z Wang C Mao J Ye A. The impact of artificial intelligence (AI) finance on financing constraints of non-SOE firms in emerging markets. Int J Emerg Markets. (2021) *ahead-of-print*. 10.1108/IJOEM-02-2021-0299

[57] Li R. Research on the impact of AI application on capital chain resilience. Eng Econom. (2023) 34:536–53. 10.5755/j01.ee.34.5.33167

[58] Chen H Zhang M Zeng J Wang W. Artificial intelligence and corporate risk-taking: Evidence from China. China J Account Res. (2024) 17:100372. 10.1016/j.cjar.2024.100372

[59] Zhong K Song L. Artificial intelligence adoption and corporate green innovation capability. Financ Res Lett. (2025) 72:106480. 10.1016/j.frl.2024.10648037907553

[60] Tseng C-J Lin S-Y. Role of artificial intelligence in carbon cost reduction of firms. J Clean Prod. (2024) 447:141413. 10.1016/j.jclepro.2024.14141340690858

[61] Song M Pan H Shen Z Tamayo-Verleene K. Assessing the influence of artificial intelligence on the energy efficiency for sustainable ecological products value. Energy Econom. (2024) 131:107392. 10.1016/j.eneco.2024.107392

[62] Wang J Liu Y Wang W Wu H. Does artificial intelligence improve enterprise carbon emission performance? Evidence from an intelligent transformation policy in China. Technol Soc. (2024) 79:102751. 10.1016/j.techsoc.2024.102751

[63] Wang J Wang A Luo K Nie Y. Can artificial intelligence improve enterprise environmental performance: evidence from China. J Environ Manage. (2024) 370:123079. 10.1016/j.jenvman.2024.123079 39490017

[64] Zhao L Ma Y Chen N Wen F. How does climate policy uncertainty shape corporate investment behavior? Res Int Business Financ. (2025) 74:102696. 10.1016/j.ribaf.2024.102696

[65] Xie Z Ali H Kumar S Naz S Ahmed U. The impact of energy-related uncertainty on corporate investment decisions in China. Energies. (2024) 17:2368. 10.3390/en17102368

[66] Nie X Chen Z Wang H Wu J Wu X Lu B. Is the “pollution haven hypothesis” valid for China's carbon trading system? A re-examination based on inter-provincial carbon emission transfer. Environ Sci Pollut Res. (2022) 29:40110–122 10.1007/s11356-022-18737-7 35112261

[67] Zhang Z Cheng S Wang C Song S Feng Y. Climate policy uncertainty and corporate investment efficiency: evidence from China. J Environ Plan Manag. (2023) 68:1–21. 10.1080/09640568.2023.227606237648195

[68] Niu S Zhang J Luo R Feng Y. How does climate policy uncertainty affect green technology innovation at the corporate level? New evidence from China. Environm Res. (2023) 237:117003. 10.1016/j.envres.2023.117003 37648195

[69] Zou X Li W Wu W Hunt A Lu H. How do executives' overseas experiences reshape corporate climate risk disclosure in emerging countries? Evidence from China's Listed Firms. Systems. (2025) 13:494. 10.3390/systems13060494

[70] Khan M. A, Meng B, Ullah I. Uncertainty and green innovation nexus: the moderating influence of ownership structure and product market competition. Corporate Social Responsibility and Environmental Management (2025) 10.1002/csr.3128

[71] Ma Y.-R, Liu Z, Ma D, Zhai P, Guo K, Zhang D, et al. A news-based climate policy uncertainty index for China. Sci Data. (2023) 10:881. 10.1038/s41597-023-02817-5 38065994 PMC10709629

[72] Chen J Xu C Li K Song M. A gravity model and exploratory spatial data analysis of prefecture-scale pollutant and CO_2_ emissions in China. Ecol Indicat. (2018) 90:554–63. 10.1016/j.ecolind.2018.03.057

[73] Yu C. -H Wu X Zhang D Chen S Zhao J. Demand for green finance: resolving financing constraints on green innovation in China. Energ Policy. (2021) 153:112255. 10.1016/j.enpol.2021.112255

[74] Baek C Baek S Glambosky M. Macroeconomic impact and stock returns' vulnerability by size, solvency, and financial distress. Financ Res Lett. (2024) 59:104718. 10.1016/j.frl.2023.104718

[75] Ng YL Lau WT Soh WN Razak NHA. Financial constraints of ASEAN firms: Impact alleviation by ESG pillars. Econ Financ Lett. (2024) 11:126–45. 10.18488/29.v11i2.3738

[76] Balan P Norden L. Are measures of corporate financial constraints universal? Evidence from Brazil. Financ Res Lett. (2024) 70:106353. 10.1016/j.frl.2024.106353

[77] Li H Bouri E Gupta R Fang L. Return volatility, correlation, and hedging of green and brown stocks: is there a role for climate risk factors? J Clean Prod. (2023) 414:137594. 10.1016/j.jclepro.2023.137594

[78] Wang X Wu W. CEO AI orientation, human resources and green innovation: an attention-based view. Kybernetes. (2024) 10.1108/K-04-2024-0964

[79] Zhao N Zhao M Long H Yuan L. Facing or evading? The impact of environmental taxes on the migration of heavily polluting enterprises in China. Environm Dev Sustain. (2025) 27:11773–96. 10.1007/s10668-023-04381-9

[80] Borenstein S Bushnell J Wolak FA Zaragoza-Watkins M. Expecting the unexpected: emissions uncertainty and environmental market design. Am Econ Rev. (2019) 109:3953–77. 10.1257/aer.20161218

[81] Campiglio E Dafermos Y Monnin P Ryan-Collins J Schotten G Tanaka M. Climate change challenges for central banks and financial regulators. Nat Clim Chang. (2018) 8:462–8. 10.1038/s41558-018-0175-0

[82] Wiersema MF Bantel KA. Top management team demography and corporate strategic change. Acad Manage J. (1992) 35:91–121. 10.5465/256474

[83] Lei L Ozturk I Murshed M Abrorov S Alvarado R Mahmood H. Environmental innovations, energy innovations, governance, and environmental sustainability: evidence from South and Southeast Asian countries. Resour Policy. (2023) 82:103556. 10.1016/j.resourpol.2023.103556

[84] Chen W Tang H He L Zhang Y Ma W. Co-effect assessment on regional air quality: a perspective of policies and measures with greenhouse gas reduction potential. Sci Total Environ. (2022) 851:158119. 10.1016/j.scitotenv.2022.158119 35987248

